# Current Perspectives on Functional Involvement of Micropeptides in Virus–Host Interactions

**DOI:** 10.3390/ijms26083651

**Published:** 2025-04-12

**Authors:** Haowen Sun, Rongrong Gu, Tingting Tang, Kul Raj Rai, Ji-Long Chen

**Affiliations:** 1 Key Laboratory of Animal Pathogen Infection and Immunology of Fujian Province, College of Animal Sciences, Fujian Agriculture and Forestry University, Fuzhou 350002, China; sunhaowen202410@163.com (H.S.); gurongrong13@163.com (R.G.); 5220623020@fafu.edu.cn (T.T.); 2 Key Laboratory of Fujian-Taiwan Animal Pathogen Biology, College of Animal Sciences, Fujian Agriculture and Forestry University, Fuzhou 350002, China

**Keywords:** sORFs, micropeptides, virus–host interactions, innate immunity

## Abstract

Micropeptides (miPEPs), encoded by short open reading frames (sORFs) within various genomic regions, have recently emerged as critical regulators of multiple biological processes. In particular, these small molecules are now increasingly being recognized for their role in modulating viral replication, pathogenesis, and host immune responses. Both host miPEPs and virus-derived miPEPs have been noted for their ability to regulate virus–host interactions through diversified mechanisms such as altering protein stability and modulating protein–protein interactions. Although thousands of sORFs have been annotated as having the potential to encode miPEPs, only a small number have been experimentally validated so far, with some directly linked to virus–host interactions and a small subset associated with immune modulation, indicating that the investigation of miPEPs is still in its infancy. The systematic identification, translational status assessment, in-depth characterization, and functional analysis of a substantial fraction of sORFs encoding miPEPs remain largely underexplored. Further studies are anticipated to uncover the intricate mechanisms underlying virus–host interactions, host immune modulation, and the broader biological functions of miPEPs. This article will review the emerging roles of miPEPs in virus–host interactions and host immunity, and discuss the challenges and future perspectives of miPEP studies.

## 1. Introduction

Micropeptides (miPEPs), also known as microproteins or small proteins, are generally less than 150 amino acids in length. They are encoded by short open reading frames (sORFs) within diverse regions of the genome that were once thought to be non-coding or lacking functional relevance to protein production [1,2,3]. The miPEP-encoded RNAs include long non-coding RNAs (lncRNAs), circular RNAs (circRNAs), pre-miRNA sequence, coding sequences (CDS), and untranslated regions (UTRs) of canonical messenger RNAs (mRNAs) [1,4,5,6,7,8]. miPEPs are derived not only from intergenic regions but also from introns, alternative reading frames within known genes, and previously unannotated regions of the genome [1,4,9]. They are expressed in both prokaryotic and eukaryotic organisms. Unlike classical bioactive peptides, miPEPs are not cleaved from precursor protein and lack an N-terminal signaling sequence [10]. Therefore, they are released in the cytoplasm immediately after translation [10]. The potential roles for functional peptides encoded by these regions was previously underappreciated, largely due to limitations in genome annotation technologies and the prevailing assumption that non-coding sequences were functionally inert [4]. However, studies of miPEPs have been getting significant attention since some critical examples were identified, such as humanin [11] and myoregulin [12], which demonstrated critical functions in diverse cellular processes. These findings underscored the importance of these small peptides in biological processes.

Recently, large-scale efforts like the ENCODE project have expanded our understanding of genomic coding sequences, revealing the diverse origins of sORF-encoded peptides. Advancements in high-throughput sequencing technologies, ribosome profiling (ribo-seq), and mass spectrometry (MS) have further confirmed that these sORFs are potentially translated into functional miPEPs with noteworthy biological relevance [1,9,13]. Emerging studies have increasingly validated hundreds of miPEPs as key regulators of cellular processes, including host immune responses to infections with pathogens [14]. For instance, a 17-amino-acid miPEP named miPEP155 (P155), encoded by the lncRNA MIR155HG, is highly expressed in inflamed antigen-presenting cells. P155 interacts with heat shock cognate protein 70 to modulate antigen presentation to T cells, thereby playing a crucial role in the initiation of the immune response [15]. Despite these advances, the field is still in its infancy. The experimental identification of sORF translation into biologically active miPEPs remains lacking. Consequently, key areas such as the systematic characterization and elucidation of the roles of miPEPs and underlying mechanisms remain to be determined.

Viruses are obligate intracellular entities with genomes composed of either DNA or RNA. To replicate and propagate, they rely on host cellular machinery [16]. In the context of virus–host interactions, increasing evidence highlights the potential roles of host miPEPs in modulating both host immune responses and viral pathogenesis [17]. On the other hand, the discovery of virus-encoded miPEPs has further expanded the scope of miPEP research, uncovering additional layers of complexity in the interaction between the host immune system and viral infection. However, little information is available about the biogenesis and function of virus-encoded miPEPs. Investigation of the virus-encoded miPEPs could offer valuable insights into viral pathogenesis and provide valuable information for the control of viral diseases. This review highlights recent advances in understanding the roles of miPEPs in virus–host interactions and discusses the challenges and future perspectives in the field of miPEP studies.

## 2. Biogenesis and Characterization of miPEPs

**Figure 1:**
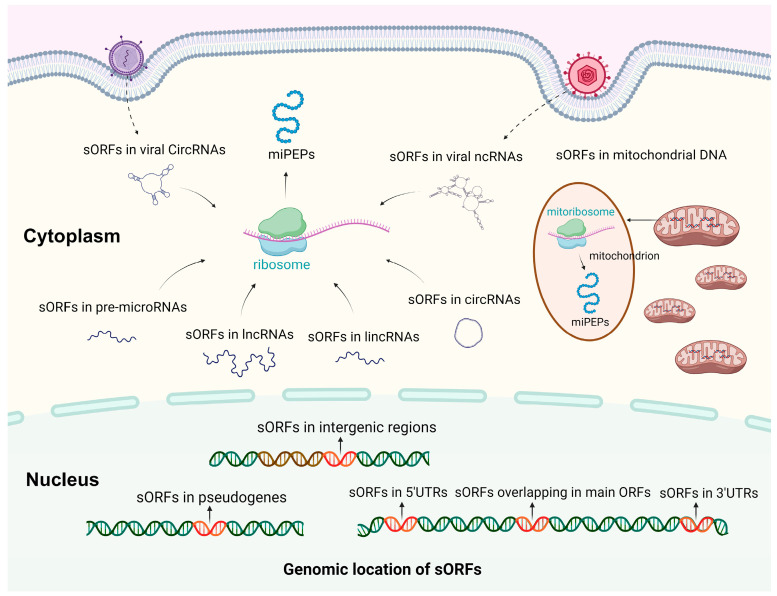
Biogenesis of miPEPs. The biogenesis of miPEPs begins with the transcription of genomic regions containing sORFs. These genomic regions include intergenic regions; pseudogenes; DNA sequences encoding the 5′ and 3′ UTRs and the CDS of mRNAs; mitochondrial DNA; as well as viral genome. Following transcription, the translation of sORFs into miPEPs can be mediated by ribosome recruitment via IRES, with further translation efficiency enhanced by m^6^A modifications. Additionally, miPEPs are also derived from functional sORFs present in various RNA molecules, including lncRNAs, circRNAs, pre-microRNAs, and lincRNAs.

The biogenesis of miPEPs begins with the transcription of genomic regions containing sORFs, which are typically found within non-coding RNAs (ncRNAs), as well as within the CDS or UTRs of mRNAs. Regulatory elements, such as internal ribosome entry sites (IRES) and N6-methyladenosine (m^6^A) methylation sites have been shown to mediate miPEP translation. IRES are RNA sequences, typically located in the 5′UTR upstream of the ORF, that facilitate translation without the need for the 5′ cap [5]. IRES elements can also be located within or between ORFs. These elements recruit ribosomes, enabling ribosome assembly and the translation of sORFs into miPEPs. For example, sORFs located in lncRNAs containing IRES elements could be translated into miPEPs [18]. Furthermore, m^6^A modifications has been shown to enhance the translation of endogenous ncRNAs, particularly circRNAs, and numerous circRNAs with translation potential have been identified [19]. It is also possible that m^6^A modifications may drive the translation of lncRNAs. These sORFs may be translated into small peptides through mechanisms that bypass traditional translation initiation, such as ribosome reinitiation or upstream open reading frame translation. Unlike full-length proteins, miPEPs are derived from functional sORFs that exist in various RNA molecules, including lncRNAs, circRNAs, pre-microRNAs, lincRNAs, and UTRs of mRNAs. sORFs found in the 5′ and 3′ UTRs are referred to as upstream open reading frames (uORFs) and downstream open reading frames (dORFs), respectively. miPEPs from both uORFs and dORFs have been shown to regulate the translation of the main CDS [20,21]. Additionally, sORFs are found in pseudogenes and intergenic regions. Interestingly, some miPEPs are also derived from DNA in cellular organelles. For example, MOTS-c, humanin, and small humanin-like peptides (SHLP) 1–6 are encoded by sORFs within mitochondrial DNA [22,23,24,25,26,27] (Figure 1).

Several approaches are available to identify sORFs and assess their translation potential into miPEPs. Commonly employed approaches include RNA sequencing (RNA-seq), ribo-seq, MS, and so forth, which are simultaneously complemented by bioinformatics analysis. These methods integrate various techniques, such as predicting ORFs, analyzing translation start elements like IRES, investigating histone modifications, and performing translation omics and proteomics profiling. Readers can refer online database for sORF identification such as sORFs.org [28], SmProt [29], OpenProt [30], ARA-PEPs [31], and so forth. However, these methods are primarily useful for identifying sORFs with the potential to produce miPEPs, rather than directly assessing their translation status and functioning. Functional characterization of sORFs and miPEPs requires experimental validation, including in vitro translation analysis to confirm protein-coding potential, development of specific antibodies for detection, ultrafiltration of cell lysates combined with mass spectrometry analysis, and advanced genetic manipulation techniques, such as CRISPR-Cas9 tagging, to explore their biological functions. However, a very large number of miPEPs encoded by sORFs remain poorly understood. Extensive studies including systematic identification, assessment of translational status, in-depth characterization, and functional analysis of sORFs and miPEPs are required in the future.

## 3. Roles of miPEPs in Virus–Host Interactions

### 3.1. Virus–Host Interactions

Virus–host interactions are highly intricate and encompass a vast array of viruses and hosts, ranging from bacteriophages infecting bacteria to human pathogens such as influenza A virus (IAV), human immunodeficiency virus, and severe acute respiratory syndrome coronavirus 2 [16]. Viruses can operate cellular machinery to complete their replication process through interacting with diverse host cell components [17]. These interactions serve two primary purposes: they either directly facilitate key steps in the viral lifecycle, such as entry, intracellular trafficking, genomic replication, gene expression, and the release of viral progenies, or they antagonize cell-intrinsic immune defenses, including type I interferon (type I-IFN)-mediated responses and various cellular pathways like apoptosis, necroptosis, pyroptosis, and ferroptosis [16,17].

The innate immune system is the first line of defense, relying on pattern recognition receptors (PRRs) to detect viral components [16,32]. Activation of these PRRs initiates signaling cascades that result in the production of IFNs and pro-inflammatory cytokines, which mediate innate immunity against viral infection [2,17]. These innate immune responses are further complemented by the adaptive immune system, which provides long-term, virus-specific immunity through the elimination of infected cells by T cells and the production of neutralizing antibodies by B cells [33]. Cellular mechanisms, such as the ubiquitin–proteasome system and autophagy, also play dual roles in terms of regulating viral replication and shaping immune responses [34,35]. Interestingly, viruses such as coronaviruses, flaviviruses, and influenza viruses exhibit species-specific interactions due to their ability to infect a range of hosts, including insects, birds, and mammals [36]. These pathogens have evolved unique mechanisms tailored to each host species likely through specifically binding to their receptor molecules on the surfaces of cells, while host antiviral responses and restriction factors further influence disease outcomes [16]. Differences in host immune responses, including the roles of antiviral restriction factors, can determine whether a viral infection leads to clearance, persistence, or severe pathogenesis in a given species [16].

Additionally, viral infections regulate the expression of numerous host genes [2], including ncRNAs [37,38,39] that may encode miPEPs through sORFs [15,37]. Some viral genomes also contain sORFs encoding miPEPs, which play critical roles in virus–host interactions and viral pathogenesis [40]. Genome-wide high-throughput methods have identified an increasing number of sORF-containing lncRNAs that are potentially involved in immune responses [13], further linking miPEPs to virus–host interplay. miPEPs contribute to the regulation of immune signaling, inflammation, and antiviral defenses by altering protein stability, modulating protein–protein interactions, and so on. On the other hand, viruses have developed sophisticated strategies to evade immune detection [16,41]. These include suppressing IFN signaling, altering antigen presentation, and hijacking host transcriptional and translational machinery to ensure successful replication and survival [41]. The battle between immune activation and viral evasion highlights the dynamic nature of virus–host interplay [41,42]. Understanding these molecular interactions is essential for developing targeted therapeutic strategies and vaccines. Recently, emerging evidence underscores the biological significance of both host and virus-derived miPEPs in virus–host interactions, especially in shaping infection outcomes. This field deserves in-depth and systematic investigation.

### 3.2. Roles of Host miPEPs in Virus–Host Interactions

Host miPEPs are encoded by sORFs within the different genomic locations. While thousands of these sORFs have been annotated using approaches such as RNA-seq, ribo-seq, and other proteomic techniques, only a limited number of sORFs have been experimentally validated as they are truly translated into miPEPs and play roles in various biological processes. In this review, we only focus on the functional involvement of these miPEPs in virus–host interactions.

**Figure 2:**
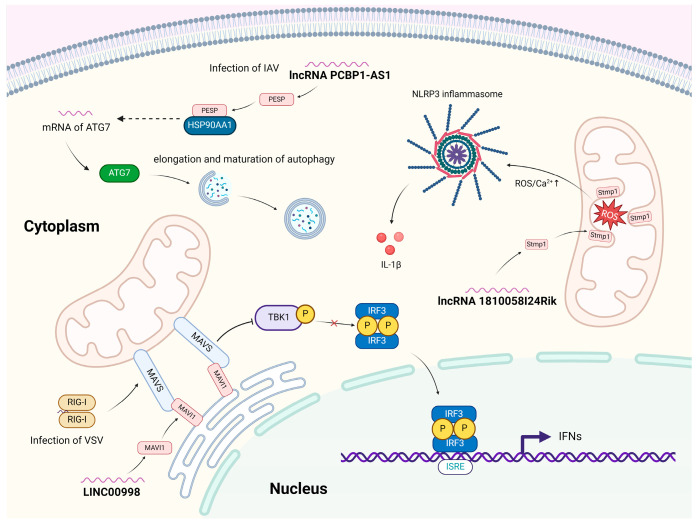
Proposed mechanism of action of some representative miPEPs (MAVI1, PESP, and Stmp1) in virus–host interactions. MAVI1, encoded by LINC00998, targets MAVS on the mitochondria. By suppressing MAVS-mediated antiviral innate immune responses, MAVI1 facilitates VSV replication. PESP, encoded by lncRNA PCBP1-AS1, is stabilized by interacting with HSP90AA1. This stabilization promotes the transcription of ATG7, key regulator of autophagy, and contributes to elongation and maturation of autophagy, resulting in enhanced IAV replication. Stmp1, encoded by lncRNA 1810058I24Rik, is localized to the inner membrane of mitochondria. It promotes NLRP3 inflammasome activation by triggering reactive oxygen species (ROS) generation and inducing Ca^2+^ flux, potentially playing a role in virus–host interactions.

miPEPs can participate in various aspects of immune responses to viral infections, from viral recognition to mechanisms underlying the action of effectors [3,20,43]. Virus-induced immune signaling activates host defense pathways, commencing with the detection of viral components such as RNA or DNA by PRRs [16]. This detection triggers signaling cascades that activate transcription factors, including nuclear factor-kappa B (NF-κB) and interferon regulatory factors 3 and 7 [41]. These transcription factors drive the production of numerous cytokines and the expression of antiviral genes, which limit viral replication and enhance immune defenses [44,45,46]. At the same time, when the virus is sensed by the host, expression of miPEPs can also be regulated by the activation of innate immune signaling mediated by the PRRs [2,15,47]. Viral infection induces the production of miPEPs, which, in turn, can modulate the innate immunity and the subsequent shaping of adaptive immune responses. Certain miPEPs act as immune modulators, inhibiting antiviral defenses [2]. For example, miPEPs can regulate various cellular pathways, including autophagy, apoptosis, necroptosis, and ferroptosis, and thereby regulate viral replication [48,49]. Recently, we showed that the miPEP named as PESP promotes IAV replication by enhancing IAV-induced autophagy through upregulation of autophagy related gene 7 (ATG7) [2]. Moreover, it has been reported that miPEP MAVI1, encoded by the ncRNA LINC00998, is downregulated during Vesicular Stomatitis Virus (VSV) infection. MAVI1 inhibits I-IFN signaling by directly binding to the mitochondrial antiviral signaling protein (MAVS) on mitochondria [50]. Additional host miPEPs and their roles in virus–host interactions are summarized in Table 1, and the proposed mechanisms of action of miPEPs, as characterized in virus–host interactions, are shown in Figure 2.

### 3.3. Roles of Virus-Derived miPEPs in Virus–Host Interactions

Some viral miPEPs derived from viral genomes are also being characterized as critical players in virus–host interplay. For instance, Bombyx mori cytoplasmic polyhedrosis virus (BmCPV) expresses vSP27 from circRNA-vSP27, which induces ROS generation, activates NF-κB signaling, promotes antimicrobial peptide expression, and suppresses BmCPV infection [51]. Similarly, vcircRNA_000048 from BmCPV encodes vSP21, a 21-amino-acid peptide that suppresses viral replication by activating the NF-κB/autophagy pathway via interaction with ubiquitin carboxyl-terminal hydrolase [52]. In Kaposi’s sarcoma-associated herpesvirus (KSHV), T3.0 RNA encodes two miPEPs, vSP-1 (48 amino acids or 48-aa) and vSP-2 (27-aa), which regulate KSHV reactivation and latency. vSP-1 interacts with the replication and transcription activator (RTA), controlling its abundance and activity, while their translation highlights polycistronic sORF expression from misannotated ncRNAs [53,54]. Additionally, ribosome profiling of human cytomegalovirus, KSHV, and Vaccinia virus has revealed numerous sORFs [55], expanding the viral coding repertoire through alternative splicing, RNA editing, and non-canonical translation (please refer Table 1 for the additional information).

**Table 1:** List of miPEPs directly involved in virus–host interactions.

Name	Size (aa)	Origin/Source	Virus–Host Interactions	Function/Mechanism	Ref.
Host miPEPs involved in virus–host interactions					
ORF-674	71	XR_001139971.3 (lnc557)	*Bombyx mori* nucleopolyhedrovirus (BmNPV)	Not Available	[56]
PESP	110	lncRNA PCBP1-AS1 (human)	IAV	Enhances the IAV-induced autophagy by increasing the expression of ATG7.	[2]
MIR22HG peptide	NA	lncRNA MIR22HG (human)	IAV	Not Available	[47]
SMIM30/ MAVI1	59	LINC00998	VSV	Endoplasmic reticulum–localized microprotein that suppresses antiviral innate immune response by targeting MAVS on mitochondria.	[50]
Virus-encoded miPEPs involved in virus–host interactions					
vsp21	21	vcircRNA_000048 (silkworm)	BmCPV	Attenuates the viral replication.	[40]
vSP27	NA	circular RNA (circRNA-vSP27)	BmCPV	Suppresses BmCPV infection.	[51]
VSP59	59	S10 dsRNA genome	BmCPV	Negatively regulates of viral replication.	[57]
PB1-F2	87–90	Influenza A/PR/8/34 virus	IAV	Mitochondria localized miPEP that induces apoptosis in host cells.	[58]
vSP-1	48	T3.0 RNA from KSHV	KSHV	Precisely control of RTA abundance and activity in KSHV reactivation and initiates the establishment of latency of the KSHV.	[53,54]
vSP-2	27	T3.0 RNA from KSHV	KSHV	Not Available	[53,54]

### 3.4. Roles of Host-Derived miPEPs in Physiological and Pathological Processes: Potential Roles in Virus–Host Interactions

Over the past decade, there have been an increasing number of studies indicating that miPEPs play critical roles in promoting or suppressing tumor growth [59,60,61,62,63,64,65,66,67,68,69,70,71,72,73,74] and other physiological and pathological processes, including apoptosis [26,27,49,75,76], stress response [49], and inflammation [43,77,78,79]. In particular, the functioning of miPEPs in the occurrence and development of tumors has attracted growing interest [6,75]. Although the role of miPEPs in the context of virus–host interactions remains largely unknown, their function in other cellular processes suggests that they are potentially involved in virus–host interactions. For example, several IAV proteins induce mitophagy [80], autophagy [81], and ferroptosis [82] to inhibit MAVS-mediated antiviral signaling [80,81,82]. By suppressing the I-IFN response, these processes facilitate viral replication and immune evasion. Therefore, miPEPs previously characterized in various cellular pathways such as mitophagy, autophagy, apoptosis, and some cancer-related signaling may also play a key role in virus–host interactions. Interestingly, several miPEPs have been shown to interact directly with MAVS [50,83]. Some of these miPEPs have been shown to modulate key antiviral signaling pathways, including STAT3 and NLRP3 inflammasome signaling [43,79,84]. Table 2 lists some representative miPEPs involved in these cellular processes.

**Table 2:** miPEPs potentially involved in virus–host interactions.

Name	Size (aa)	Origin/Source	Function/Mechanism	Ref.
ASRPS	60	LINC00908	miPEPs regulate innate or adaptive immunity.	[15,84,85,86,87]
P155	17	lncRNA MIR155HG		
miPEP31	44	pri-miRNA-31		
Stmp1/Mm47	47	lncRNA 1810058I24Rik	Activates the NLRP3 inflammasome pathway	[43,77,78,79,88]
SHLP2	26	mitochondrial 16S rRNA gene	Regulate apoptosis.	[26,27,49,75,76]
PIGBOS	54	PIGB opposite strand 1		
FORCP	79	LINC00675		
YY1BM	21	LINC00278		
AC115619-22aa	22	lncRNA AC115619	Regulates autophagy	[48]
PINT87aa	87	LINC-PINT	Regulates mitophagy	[89]
PACMP	44	lncRNA CTD-2256P15.2	Modulates DNA damage response.	[90]

## 4. Challenges and Future Perspectives of miPEP Study

Despite the exciting potential of miPEPs in virus–host interactions, several challenges hinder their study. One major difficulty lies in detecting and characterizing these small peptides due to their typically low expression levels and small sizes [91,92,93,94]. Conventional proteomics methods often struggle to differentiate miPEPs from background noise, necessitating more sensitive approaches such as ribo-seq and MS with enhanced detection capabilities [95,96,97,98]. Immunoblotting remains a traditional and straightforward technique for detecting proteins, including small peptides. However, generating specific antibodies against miPEPs presents significant hurdles. Peptides containing transmembrane domains may restrict the availability of epitopes suitable for antibody production, complicating detection and validation efforts. Moreover, the lack of comprehensive databases and annotation for miPEPs further challenges their identification and functional characterization.

Another challenge is the dynamic nature of their expression, which varies with cellular contexts, stress conditions, and viral infections [2,15,75]. Understanding how miPEPs are regulated during infections requires integrative transcriptomic and proteomic analyses, as well as functional validation in relevant biological models. Furthermore, while in vitro systems provide valuable insights, studying miPEPs in complex tissue environments is essential for understanding their roles in vivo.

Addressing these challenges will require advancements in detection methodologies, computational prediction tools, and functional assays. Overcoming these obstacles will not only improve our understanding of miPEPs in viral infections but also open new avenues for therapeutic interventions targeting these small yet potentially influential molecules.

Although progress has been made in understanding the role of miPEPs in virus–host interactions, the functional relevance of these small peptides in viral pathogenesis still remains elusive. This is an area worth exploring further. The fact that hundreds of lncRNAs, each containing lots of sORFs, are polyadenylated, localized in the cytoplasm, and associated with ribosomes further supports the notion that the translation of miPEPs may be a widespread phenomenon [99,100,101]. However, the extent to which these miPEPs contribute to biological processes needs to be further determined.

One promising avenue for future research is the systematic identification and characterization of the miPEPs that influence viral replication and immune evasion. Recent studies have demonstrated that viral infections, including IAV, can alter sites of translation initiation, sometimes leading to the production of novel immune epitopes [102]. Ribo-seq and translation initiation mapping in infected cells could help uncover miPEPs that modulate antiviral responses. Since IAV infection is known to induce mitophagy, autophagy, and ferroptosis while inhibiting MAVS-mediated antiviral responses, investigating whether miPEPs interact with these pathways could provide new insights into viral pathogenesis and host defense mechanisms [80,81,82].

Additionally, expanding research beyond in vitro cell culture systems is crucial. While virus–host interactions are often studied in single-cell models, infections occur in complex tissue environments that include immune and non-immune cells, extracellular matrix components, and a dynamic immune microenvironment. Advanced methodologies such as spatial transcriptomics and single-cell proteomics could be leveraged to profile miPEP expression and function in infected tissues. This would provide a more comprehensive understanding of their roles in vivo.

Ultimately, uncovering the functional significance of miPEPs in virus–host interactions could open new avenues for antiviral therapeutics. By identifying miPEPs that regulate key immune pathways, researchers may discover novel targets for intervention. The integration of high-throughput sequencing, proteomics, and advanced computational analyses will be essential in deciphering the full impact of miPEPs on viral infections and host immunity. As this field evolves, a deeper understanding of miPEPs may transform our approach to antiviral strategies and immune modulation.

## 5. Summary

miPEPs are promising yet understudied molecules implicated in various biological processes. Increasing reports have shown that they play critical roles in virus–host interactions, especially in the regulation of immune responses. However, direct evidence remains limited, emphasizing the field’s early stage. Advancing technologies like ribo-seq, proteomics, and spatial transcriptomics, coupled with experimental validation, will be key to uncovering their functions in viral infection. Moreover, future research should explore miPEPs as therapeutic targets, paving a way for the development of new antiviral strategies. In summary, better understanding of their roles in virus–host interactions could deepen our knowledge of viral pathogenesis and drive targeted interventions for viral infectious diseases.

## Data Availability

No new data were created or analyzed in this study. Data sharing is not applicable to this article.

## Abbreviations

The following abbreviations are used in this manuscript:

HSP90	Heat shock protein 90
NLRP3	NOD-like receptor family Pyrin domain-containing protein 3
LincRNAs	Long intronic non-coding RNAs
CRISPR-Cas9	Clustered Regularly Interspaced Short Palindromic Repeats/CRISPR-associated protein 9
